# Bioactive Element Biodistribution of Different Biological Substrates in Sheep and Goats

**DOI:** 10.3390/ani15121686

**Published:** 2025-06-06

**Authors:** Vincenzo Nava, Francesca Aragona, Angela Giorgia Potortì, Salvatore De Caro, Beatrice Di Bella, Federica Litrenta, Francesco Fazio

**Affiliations:** 1 Department of Biomedical, Dental and Morphological and Functional Imaging Sciences (BIOMORF), University of Messina, Viale Palatucci, 13, 98168 Messina, Italy; vincenzo.nava@unime.it (V.N.); angela.potorti@unime.it (A.G.P.); federica.litrenta@unime.it (F.L.); 2 Department of Veterinary Science, University of Messina, Via Giovanni Palatucci, 98168 Messina, Italy; ffazio@unime.it; 3 Department of Engeneering, University of Messina, C/da di Dio (S. Agata), 98166 Messina, Italy; salvatore.decaro@unime.it (S.D.C.); beatrice.dibella@unime.it (B.D.B.)

**Keywords:** small ruminants, bioactive elements, biological biomarkers, biomonitoring, One Health

## Abstract

The production and reproduction of small ruminants, such as sheep and goats, living under extensive range-grazing conditions may be influenced by the availability of nutrients and the mineral content of forage. This study evaluated the biodistribution of calcium (Ca), copper (Cu), iron (Fe), magnesium (Mg), manganese (Mn), sodium (Na) and zinc (Zn) in whole blood, serum, blood clots, plasma, plasma sediments and hair in sheep and goats. Forty clinically healthy, nonpregnant female sheep and goats were enrolled in the study. Hair and blood samples were performed in duplicate to assess mineral concentration in biological substrates using a Thermo Scientific iCAP- Q ICP-MS spectrometer (Omaha, Ne, USA). Higher levels of Ca, Cu, Fe, Mg, and Na were observed in blood and blood clots than in serum, plasma, plasma sediments and hair in sheep and goats. A significant effect of species was observed for all the bioactive element concentrations in the investigated substrates (*p* < 0.05). The results revealed positive correlations between serum and plasma for all investigated elements in sheep (*p* < 0.0001) and for Mn and Na in goats (*p* < 0.001), as well as between serum and blood for Ca, Fe, Mg and Mn in sheep (*p* < 0.0001) and for Ca and Mn in goats (*p* < 0.001). Plasma and blood showed a positive correlation for Ca, Fe, and Mn (*p* < 0.001) in sheep, and for Fe, Mn, Na, and Zn (*p* < 0.0001) in goats. These findings elucidate differences in biodistribution between sheep and goats, offering valuable insights for livestock production.

## Simple Summary

In the context of biomonitoring, it is essential to examine the mineral concentration in small ruminants and the environmental backdrop that may influence their well-being, productivity, and reproduction. Bioaccumulation of bioactive elements such as calcium, copper, iron, magnesium, manganese, sodium and zinc in sheep and goats was investigated using biological substrates (whole blood, serum, blood clot, plasma, plasma sediment, and hair) so that the differences in bioaccumulation in different substrates and possible variations between species could be understood. Based on the results obtained, hair samples do not appear to be a valid alternative to blood, plasma, and serum samples for biomonitoring bioactive elements in sheep and goats. The present results indicated that the biodistribution of the investigated elements in serum and plasma had lower values than in whole blood, probably due to the higher water content. Our study sheds light on environmental biomonitoring through “sentinel” animals such as sheep and goats, providing useful information for livestock production.

## 1. Introduction

Essential mineral elements include certain macro-minerals and trace elements that are necessary for the functioning of biological systems in animals [1]. These bioactive elements participate in the various defense mechanisms of the body, including the immune system and defense against oxidative stress. They are also necessary parts of vitamins, lipids, proteins, and even carbohydrates [2,3]. In addition, they are used as enzyme activators or in the production of metal enzymes involved in processes such as cellular homeostasis, electron transport and bone metabolism. Bioactive elements are as essential in the diet as proteins or energy. It is necessary to measure and adjust their intake [4]. In fact, an excess or a deficiency can affect the health and productivity of livestock [5,6]. Bioactive element deficiency causes biochemical malfunction, irregular bodily processes, and diseases [5,6]. In particular, a deficiency of calcium, magnesium, iron, zinc and phosphate can result in bone fractures, cardiovascular incidents, and neuromuscular impairment. Conversely, excessive mineral intake can have significant health repercussions. For example, hypercalcemia can lead to nephrolithiasis, cardiac arrhythmias and soft-tissue calcification, while excessive iron accumulation can cause oxidative stress and damage organs and tissues [7,8]. In small ruminants, sodium (Na^+^) plays an important role as a of bioactive element. It is the primary positively charged ion found in the fluid between cells and plays numerous roles in the body. It facilitates the uptake of chlorine, amino acids, glucose, and water, and serves as an accessory in several transportation systems. Sodium plays a crucial role in facilitating the transportation networks that are essential for neuromuscular activity, neuronal function and the regulation of body temperature. Along with potassium (K^+^) and chloride (Cl^−^), sodium plays a vital role in regulating the body’s pH and osmotic pressure. Most of the sodium in the body is found in body fluids and bones [9]. The typical sodium concentration in blood plasma ranges from 3.2 to 3.5 mg/L in ruminants [10]. Blood plasma sodium concentration only decreases when an animal has a severe shortage [8]. Insufficient sodium consumption leads to a decrease in the volume of blood plasma and interstitial fluid in an animal [11,12]. Sodium is primarily excreted from the body through urine, sweat, and milk. It is absorbed in the rumen, omasum, and intestines of ruminants, resulting in an average absorption rate of 91%. However, these rates can range from 85% to 98%. Small ruminants experience increased fecal dry matter content due to this absorption advantage. Others macro-minerals, such as calcium (Ca), play a fundamental role in bone tissue and muscle contraction, and are required for growth and milk production [13]. Bone serves as the repository for calcium, housing approximately 98% of the body’s total calcium content. It is a highly dynamic organ that undergoes continuous metabolism and remodeling throughout its life since it is essential for providing support, maintaining mineral equilibrium, and safeguarding soft tissues in the body [13].

Mineral nutrients are essential for the health, growth, and production of all food-producing animals. Magnesium (Mg) is regarded as one of the seven key macro-minerals in farm animal diets. It is essential for numerous physiological functions in the animal body, such as enzymatic processes and the normal functioning of the nervous system, and its absence leads to various dysfunctions. Magnesium is a vital dietary component for the growth and survival of animals. It plays a crucial role in cellular metabolism and bone growth. Oxide, carbonate, and sulfate are highly bioavailable sources of magnesium for livestock [14]. Some elements are important for rumen functions in ruminants. Among these are iron (Fe), copper (Cu), and manganese (Mn), which is responsible for digestion and fertility. Copper enhances the functionality of immune cells such as leukocytes, lymphocytes and neutrophils. It also plays a vital role in regulating progesterone production by luteal cells through its involvement with superoxide dismutase. Iron and manganese are essential components of catalase, peroxidase, and cytochrome oxidase in the fight against oxidative stress.

In dairy cattle, plasma iron concentrations decrease during the acute phase response to immunological challenges, whereas plasma copper concentrations may increase [2]. Iron and manganese contribute to ovarian function and estrogen and progesterone production by acting as cofactors in cholesterol synthesis, which is a precursor for steroids such as estrogen and progesterone [15]. Copper and iron are vital for thyroid hormones due to their role in synthesizing or transforming these hormones. Copper deficiency impedes the secretion of tyrosine hydroxylase and dopamine beta enzyme systems, both of which require copper, in hypothalamic neurons. This impedes the synthesis of thyroid hormone-releasing factor. Copper-dependent peroxidase enzyme in the thyroid gland also suppresses the release of thyroid hormones [16].

This investigation aimed to assess the biodistribution of Ca, Cu, Fe, Mg, Mn, Na, and Zn in six different biological substrates (whole blood, serum, blood clots, plasma, plasma sediments, and hair) in two small ruminant species (sheep and goats). This study could contribute to our understanding of the biodistribution of bioactive elements that regulate biological processes and provide health benefits in animal production. This is because both species have similar physiological aspects. However, as they feed on different botanical species, they could exhibit different bioaccumulation patterns based on their feeding behaviors. Additionally, possible correlations in element concentrations across the various biological substrates were examined for each species.

## 2. Materials and Methods

### 2.1. Experimental Design

All housing and care conformed to the standards recommended in the Guide for the Care and Use of Laboratory Animals and in Directive 86 of 609 CEE. The experimental protocol has been reviewed and approved by the standard recommended by the Ethical Committee of the University of Messina (06/2023).

A total of 20 clinically healthy, non-pregnant female sheep (Barbaresca breed, 2-year old; mean body weight, 40 ± 1 kg) and 20 non-pregnant female goat (Sicilian native breed, 3-years old; mean body weight, 35 ± 1 kg) were used. Prior to the experimental protocol, each animal’s physiological parameters, rectal temperature (38.1–39.3 °C), respiratory rate (18–32 rpm), and heart rate (68–92 bpm) were monitored to evaluate their health status. The animals were fed on semi-extensive pastures consisting of silt soil, hilly ground, and a permanent polyphytic pasture made up of pasture meadows and cut grain crops (such as cereals and legumes on suitable soils). The pastures were based on perennial forage (2–3 years) that self-seeded. Based on the preliminary monitoring of the animals’ good health status at physiological and hematological levels and weight, no feed supplements were used except during lactation in both species, confirming that the type of grazing was sufficient for the subjects considered. The pasture area contained native plant species and trees such as olive (*Olea europaea*) and chestnut groves (*Castanea*), and was the same for both species. Animals from both species were kept on free grazing with ad libitum feed and water. Biological samples were collected during health monitoring under the supervision of the animal welfare manager and a veterinary doctor from the University of Messina.

Blood samples were collected in duplicate at 09:00 in March from jugular veins using 3.5 mL sterilized venoject tubes containing the ethylenediamine tetra acetic acid anticoagulant (K3EDTA, Terumo Corporation, Tokyo, Japan). One sample was collected with anticoagulant and one without. The samples containing K3EDTA were stored at 4 °C, and duplicates were centrifuged with samples without anticoagulant (1308× *g* × 10 min) to obtain serum and plasma. These were then stored at −20 °C until analysis. Hair was collected from the rump area (30± mm) using plastic scissors to avoid contamination from other minerals and stored in plastic bags [16,17].

During sampling, environmental parameters such as ambient temperature (18 °C), relative humidity (87%), and ventilation speed (5 km/h) were monitored using a multiparametric probe (Testo 400 SE & Co. KGaA, Titisee-Neustadt, Germany). Once the values had been read through the relevant measuring probes, the continuously recorded values were synchronized and analyzed through specific software (TESTO DataControl Vv 28.13.7) and displayed and reported as an average value [18,19].

### 2.2. Sample Preparation

Sample preparation followed the procedures outlined by Dogan et al. (2024) and Nava et al. (2024) [20,21]. In brief, to determine the concentrations of mineral elements, a sample pretreatment step consisting of mineralization or acid digestion was performed using a Milestone Ethos 1 microwave digestion system (Sorisole (BG)—Italy). For the goat and sheep hair samples, 0.5 g of hair was digested with 7 mL of 65% nitric acid (HNO_3_) and 2 mL of 30% hydrogen peroxide (H_2_O_2_). The temperature was initially increased to 200 °C for 10 min at a microwave power of 1200 W and then held at 200 °C for 12 min at the same power. The amount of blood, blood clot, serum, plasma, and plasma sediment used for mineralization was also the same (approximately 0.5 g). However, the reagent volumes were 8 mL of 65% nitric acid (HNO_3_) and 2 mL of 30% hydrogen peroxide (H_2_O_2_). The operating conditions this time were as follows: (1) 2 min at 0–85 °C and 1000 W; (2) 4 min at 85–135 °C and 1000 W; (3) 5 min at 135–230 °C and 1000 W; (4) 12 min at 230 °C constant and 1000 W.

After digestion, all samples were cooled for 20 min, diluted with ultrapure water to a volume of 25 mL, filtered through 0.45 μm filters, and analyzed by ICP-MS.

The analytical blanks and the certified matrices were prepared in the same way as the corresponding samples.

### 2.3. Multielement Analysis by ICP-MS

Mineral concentration in all samples was determined using iCAP-Q ICP-MS (Thermo Scientific, Waltham, MA, USA) to analyze the following elements: ^23^Na, ^24^Mg, ^40^Ca, ^55^Mn, ^56^Fe, ^63^Cu, ^66^Zn. The operating conditions of ICP-MS have been reported previously [20,21].

The following stocks of single-element standard solutions were used for the ICP-MS analysis: Ca, Cu, Fe, Mg, Mn, Na, and Zn (1000 mg/L in 2% nitric acid) purchased from Fluka (Milan, Italy). A calibration curve using seven points was built considering a concentration included between 10 and 200 μg L^−1^ for Cu, Fe, Mn, and Zn; and between 0.1 and 5 mg/L for Ca, Na, and Mg.

A stock standard solution of ^45^Sc, ^73^Ge, ^115^In, and ^209^Bi at 1000 mg/L in 2% nitric acid (Fluka, Milan, Italy) was used to prepare an on-line internal standard solution (1.5 mg/L), to correct for matrix deviation and instrumental drift.

For the ICP-MS calibration, a solution containing 1 μg L^−1^ of ^7^Li, ^59^Co, ^138^Ba, ^209^Bi, ^142^Ce, ^115^In, and ^238^U in 2% nitric acid and 0.5% hydrochloric acid, purchased from Thermo Fisher Scientific (Bremen, Germany), was used.

### 2.4. Analytical Performances

To validate the method in terms of linearity (R^2^), sensitivity (LOD and LOQ), and accuracy (% of recovery), two certified matrices were used: Whole Blood Metals Control Level 3 for blood, blood clot, serum, plasma, and plasma sediment samples and ERM-DB001 (human hair) for goat and sheep hair samples (Table 1). The linearity range was acceptable for the elements analyzed (R^2^ > 0.9980) (Table 1). The method also showed good sensitivity, expressed as Limit of Detection (LOD) and Limit of Quantification (LOQ) values, calculated as 3.3 σ/b and 10 σ/b, respectively, where σ is the standard deviation of the analytical blank (*n* = 6) and b is the slope of the calibration curve. Specifically, the LOD and LOQ ranged from 0.001 to 0.003 mg/kg (for Mn, in Whole Blood Metals Control Level 3) and from 1.255 to 4.142 mg/kg (for Na, in Whole Blood Metals Control Level 3) (Table 1). The lowest and highest mean recoveries were observed for Na (90.17%) in Whole Blood Metals Control Level 3 and Mn (99.67%) in ERM-DB001 (Human Hair) (Table 1).

### 2.5. Statistical Analysis

All statistical analyses were performed using the SPSS 13.0 software package for Windows (SPSS Inc., Chicago, IL, USA). First, exploratory and descriptive analyses of the data were carried out. Since not all the assumptions for using the parametric tests were satisfied, the Kruskal–Wallis and Mann–Whitney U non-parametric tests were used to test the differences among the element concentration levels of the six substrates within the same species, and to test the differences between the two species for each substrate, respectively. Significance level for statistical tests was α = 0.05. In addition, Principal Component Analysis (PCA) was carried out for both species to obtain correlations among variables and to assess the differences among the analyzed biological substrates, based on element levels. Further PCAs were carried out on each substrate to assess whether it was possible to correlate the samples analyzed with the species from which they were taken, again based on elemental concentrations. Linear regression analysis and Pearson’s correlation coefficient were used to examine the relationships between all the elements in all biological substrates.

## 3. Results

### 3.1. Element Contents in Different Biological Substrates of Sheep and Goat

As shown in Table 2, significant differences were noted for all element concentrations among the various biological substrates (*p* < 0.05). Sheep and goats had significantly higher levels of Ca, Cu, Fe, Mg and Na in their blood and blood clots than in their serum, plasma, plasma sediment and hair. In sheep, Mn levels were significantly higher in blood, blood clots, and hair; in goats, they were only significantly higher in hair. Sheep had significantly higher Zn levels in blood, blood clots, plasma and plasma sediment, whereas goats had higher contents in blood, blood clots and plasma sediment.

Statistical differences between sheep and goats were observed for all substrates in analyzed elements (*p* < 0.05). In addition, 4–6 (out of 7) elements were present in significantly different amounts (*p* < 0.05) in sheep and goat samples: four for plasma and plasma sediment (Ca, Cu, Mg and Na); five for blood (Ca, Cu, Fe, Mn and Na); and six for serum and hair (Ca, Cu, Fe, Mn, Na and Zn) and blood clot (Ca, Cu, Fe, Mg, Na and Zn).

### 3.2. Principal Component Analysis

#### 3.2.1. On Sheep Data Set

**Figure 1:**
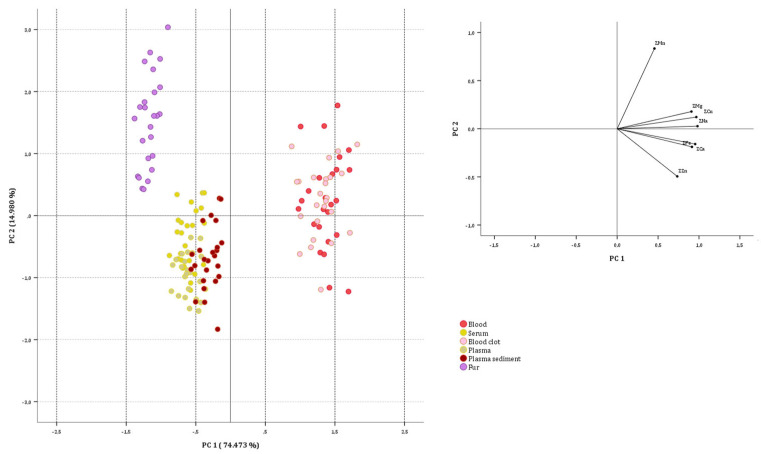
Two-dimensional score plots for the 150 sheep samples categorized as biological substrate.

To obtain correlations among the variables and to assess the differentiations among the analyzed biological substrates based on element levels, Principal Component Analysis (PCA) was applied to the normalized data. The initial multivariate matrix comprised 150 cases (sheep samples analyzed) and seven variables (Ca, Cu, Fe, Mg, Mn, Na and Zn concentrations). Prior to applying PCA, the suitability of the data for factor analysis was checked. The Kaiser–Meyer–Olkin sampling adequacy measure yielded a value of 0.867, which is above the recommended value of 0.700. Bartlett’s sphericity test showed an approximate chi-square of 1537.099, which was statistically significant at a p-level below 0.001. The matrix was therefore factored and found to be appropriate for PCA. The highest positive correlations were observed between Na and Cu (0.979), Na and Ca (0.954), and Cu and Ca (0.910). No negative correlations were observed. Two principal components (PCs) were extracted, with self-values greater than one (5.213 and 1.049) following the criterion of Kaiser. These cumulatively explained 89.453% of the total variance (74.473% and 14.980%, respectively). The communality values were always greater than 0.784. No low saturated variables were identified. The first component was positively correlated with Na, Cu, Ca, Fe, Mg and Zn (with scores of 0.981, 0.967, 0.953, 0.914, 0.908 and 0.734, respectively), while the second component was positively correlated with Mn (with a score of 0.834) and negatively correlated with Zn (−0.495). As shown in Figure 1, three well-defined primary groups were recognized. PC2 splits the blood and blood clot samples from the rest of the biological substrate. The former always exhibited positive PC1 values due to significantly higher Na, Cu, Ca, Fe, and Mg content. Regarding samples with negative PC1 values, good separation can be observed between the hair, serum, plasma, and plasma sediment groups. Hair samples had positive PC2 values, while serum, plasma, and plasma sediment samples had negative PC2 values. The characteristic that hair samples had in common was the highest Mn content and the lowest Zn content. This clearly shows the different elemental accumulations in various sheep biological substrates.

#### 3.2.2. Goat Data Set

PCA was again used to try to discriminate between biological substrate samples obtained from goats. The starting multivariate matrix constituted 120 cases (goat samples under analysis) and seven variables (Ca, Cu, Fe, Mg, Mn, Na and Zn concentrations). The factorability of the correlation matrix has been checked and achieved again (KMO value equal to 0.893; approximate chi-square value equal to 1220.653, with *p* < 0.001). The highest positive correlations were observed between Na and Ca (0.976), Na and Cu (0.935), and Cu and Ca (0.926), while the highest negative correlations were observed between Zn and Mn (−0.512), Mn and Ca (−0.455) and Na and Mn (−0.454). Two PCs with eigenvalues exceeding one (5.515 and 1.141) were extracted according to the Kaiser criterion. The extracted components explained up to 91.417% of total variance (78.787 and 12.629%, respectively). Low-saturated variables were not identified. Communality was always greater than 0.855. The first component has the highest positive correlation with Na, Ca, Cu, Zn, Mg and Fe (scores at 0.982, 0.975, 0.960, 0.920, 0.900 and 0.879) and negative correlation with Mn (−0.499), while the second component has the highest positive correlation with Mn (0.859).

**Figure 2:**
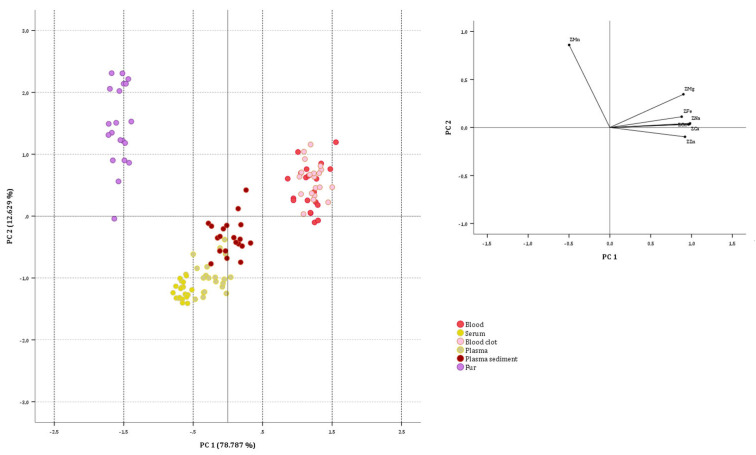
Two-dimensional score plots for the 120 goat samples categorized as biological substrate.

As can be seen in Figure 2, the blood and blood clot samples have more positive PC1 values than others and are characterized by significantly higher Na, Cu, Ca, Zn, Fe, and Mg contents. Hair samples attested the highest negative loading values on PC1, confirming that their characteristic was the highest content of Mn (the only element having the largest negative coefficients in PC1) and the lowest content in Na, Cu, Ca, Zn, Fe and Mg (the six elements having the largest positive coefficients in PC1). This clearly shows the different accumulations of elements in different biological substrates of the goat.

#### 3.2.3. On Biological Substrate Data Sets

**Figure 3:**
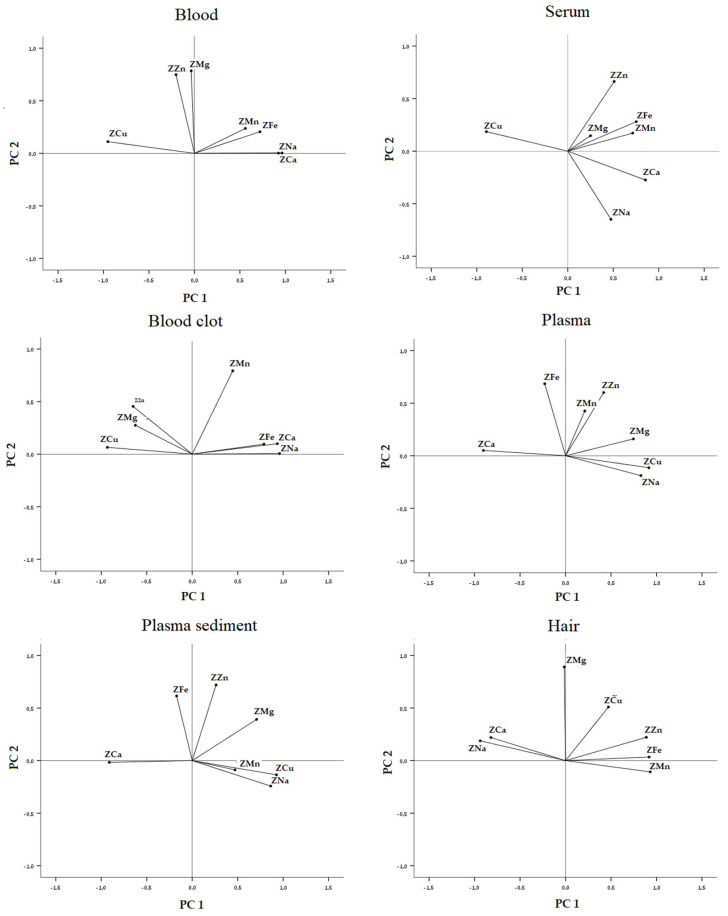
Loading plots of elements in the spaces defined by PC1 and PC2 for sample correlation to the species.

**Figure 4:**
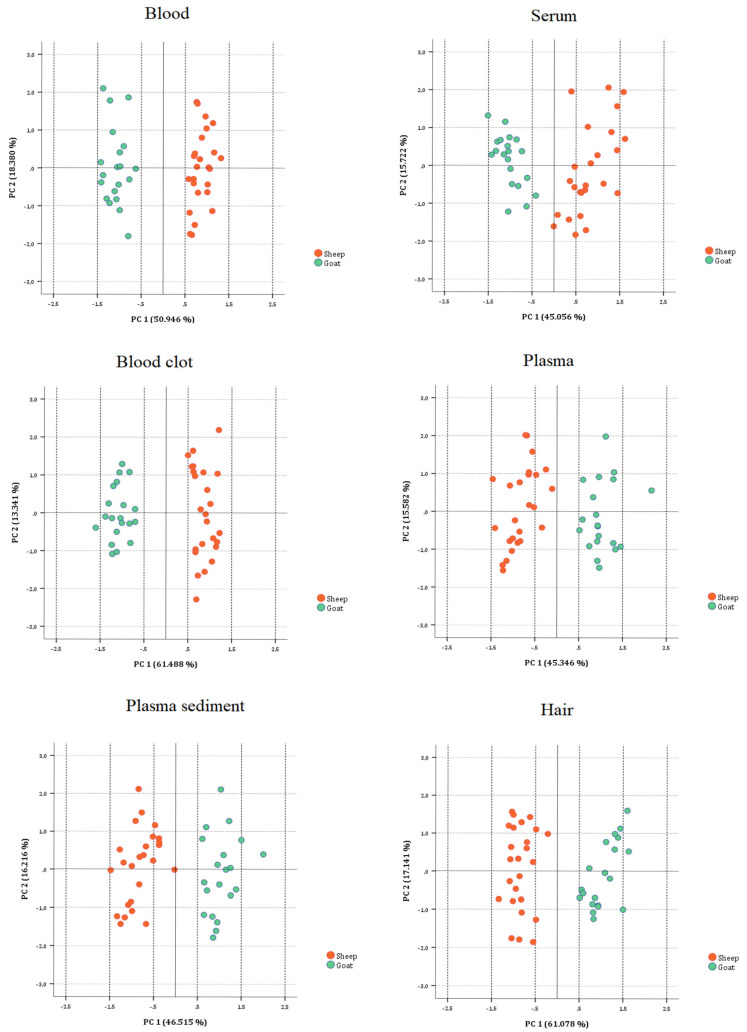
Two-dimensional score plots for PC1 and PC2 showing the split between the sheep and goat biological substrates.

For each biological substrate evaluated in this study (whole blood, serum, blood clot, plasma, plasma sediment, and hair), the PCA performed on six data sets (each with seven variables and 45 cases) showed that the categorization between sheep and goat samples was achieved. Each correlation matrix was factored and appropriate for PCA (KMO test values at 0.757, 0.737, 0.856, 0.746, 0.725 and 0.846; approximate chi-square values at 205.564, 116.382, 234.007, 129.186, 145.511 and 232.065, with a statistical significance at p-level below 0.001). Two principal components were extracted all the time with the percentages of the total variance at 69.326, 60.778, 74,829, 60.928, 62.731 and 78.219%. The loading and the score plots for the six PCA are reported in Figure 3 and Figure 4, respectively.

### 3.3. Correlation Analysis

**Figure 5:**
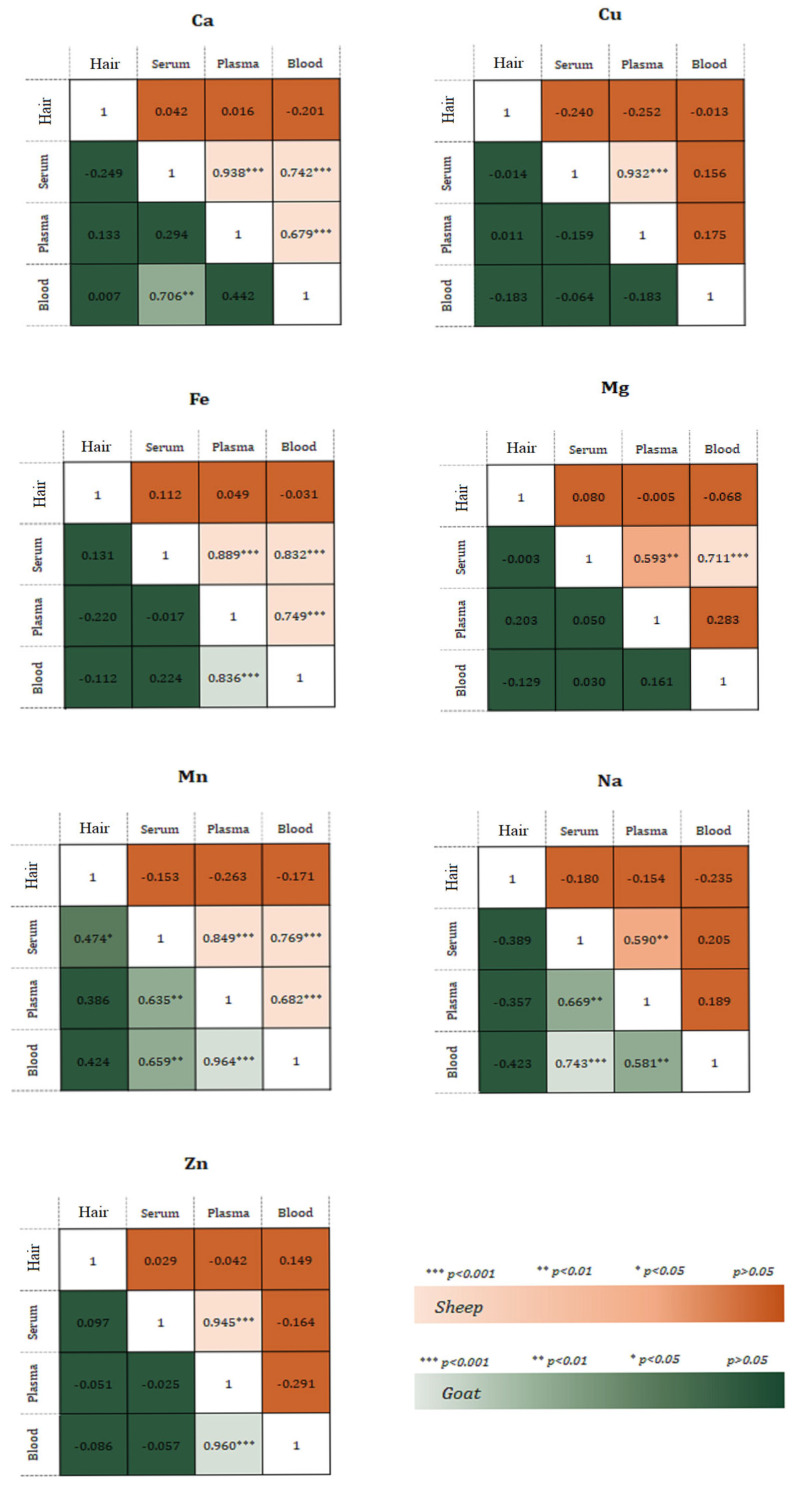
Linear correlation of Ca, Cu, Fe, Mg, Mn, Na, and Zn concentrations in the different substrates. Each heatmap shows Pearson’s correlation coefficient. * *p* < 0.05; ** *p* < 0.01; *** *p* < 0.001.

Pearson’s correlation was applied to evaluate the correlation among the different biological substrates, especially between serum and plasma element levels and between hair element levels and serum, plasma and blood element levels as shown in Figure 5. The analysis of sheep data revealed positive correlations between serum and plasma for all investigated elements, between serum and blood for Ca, Fe, Mg and Mn, and between plasma and blood for Ca, Fe and Mn.

Less correlation was observed in the goat data. There was no significant correlation for Cu and Mg among the different substrates. Only one significant correlation (positive) was found for Ca, Fe, and Zn concentrations (blood/serum and blood/plasma). The concentrations of Mn and Na in blood/serum, blood/plasma, and plasma/serum were positively correlated.

No significant correlations were found for the elements studied between hair samples and serum, plasma, and blood samples in either species. The only exception was Mn in goats (positively correlated with hair/serum).

## 4. Discussion

The assessment of bioactive element bioaccumulation by monitoring biological substrates, indicating both acute and chronic storage, is a useful and innovative approach for evaluating the environmental status in which small ruminants live. This study allows us to assess not only the environmental component but also the effect that the type of environment according to its characteristics has on the physiological state of animals in production. It is essential to prevent deficiencies, excesses, and nutritional disorders while also promoting animal welfare [22,23]. Blood samples are usually used to determine the status of macro-minerals and trace elements as they are good indicators of bioaccumulation [19]. Higher concentrations of Ca, Cu, Fe, Mg, and Na were observed in the blood and blood clots of both sheep and goats compared to the other substrates investigated. Although there have been a number of studies on this subject in the literature, no real reference values have been found for the species [24,25]. In fact, the concentration of bioactive elements could be influenced by many factors, such as lactation, pregnancy, age, breed, pasture, and environment [26]. The concentrations in sheep and goats were compared to values obtained from the literature. Calcium is crucial for animal homeostasis, encompassing coagulation, mineralization of bones and teeth, hormone secretion and brain excitability [27]. The Ca concentration obtained in the present study was significantly higher in the blood of sheep and goats than in their hair, compared to other analyzed substrates. The Ca concentration in sheep blood obtained in the present study was similar to that reported in the literature for Californian sheep [28]. However, it was lower than the concentration reported by Schweinzer et al. (2017) in serum samples of sheep and goats using the same analytical technique [29]. The present results were comparable with a previous study that analyzed serum Ca concentration in goats monitored during mild lactation, using the same analytical method [27]. Copper levels were higher in the blood and blood clots of sheep and goats, and lower in goat hair. The obtained serum Cu levels were consistent with reports by other authors using the same analytical method [27,29,30,31]. These bioactive elements affect bone density and oxidative stress. Copper concentration in the diet is known to vary depending on soil level, drainage conditions and herbage species, for example, seeds, and seed byproducts are high in copper, while straw has virtually none [32]. The same bioactive elements results were comparable with the highest concentration observed in blood than other biological substrates in horses [33,34,35,36]. The different blood components can indeed accurately reflect the metabolic state of animal tissues in order to assess tissue damage, disturbances in organ function, adaptation of the animal to nutritional and physiological challenges, and specific metabolic or nutritional imbalances [37]. In fact, mineral concentration in blood rapidly changes in response to physiological status; it is responsible for acute short-term changes, and keratinized structures respond more slowly, allowing for long-term assays [38].

Hair can be easily collected and stored for some time; furthermore, trace elements are accumulated in hair in higher concentrations than in blood [33]. Concentrations of bioactive elements in hair, however, are biased by breed, age, gender, color of the hair, and contamination [39]. The use of hair to investigate the mineral profile is relatively recent in small ruminants and scarce data are available for comparison [24].

Difference in mineral concentration in sheep and goat hair could be considered as the result of the natural preference of the animal for a specific forage, rich with these components [39]. The use of this indicator can be very advantageous, as its prevalence is high and the assessment of this indicator is quick, practical, and feasible to be applied on-farm. In fact, previous studies in goats showed the possible association between trace element concentrations in the environment and in hair [25]. Fe, Na, and Zn concentration obtained in the present study resulted to be significantly higher in sheep and goat blood substrate and lower in sheep and goat hair compared to other analyzed substrates. Goat blood concentration of Zn was lower to that reported in the literature [30]. Zinc is affected from geographical area. In addition to this, food process, dietary interactions, drug interactions, genetic disorders, and diseases affect blood serum element levels as well [39]. It is an essential element for the animal and human organism. It is involved in all physiological and regulatory processes and is necessary for the metabolic activities of numerous metalloenzymes [21]. Mg levels were higher in blood and blood clot substrate in sheep and goat. Mn concentration in blood resulted higher in blood and lower in plasma substrate. Plasma and plasma sediment, as well as blood clot, has never been used as a bioaccumulation substrate for bioactive elements in domestic animals. However, both substrates have already been observed in humans for the assessment of heavy metal bioaccumulation and as a biomaterial use [8,40,41]. A different biodistribution of Ca, Cu, Fe, Mg, Mn, Na, and Zn in each substrate was observed between the investigated species. The concentration of Ca in each substrate was greater in sheep than in goats, whereas the concentration of Cu in each substrate was higher in goats than in sheep. Fe and Mn levels reported in blood, serum, and blood clot was higher in sheep compared to goats, whereas higher concentration in hair was observed in goats compared to sheep. Compared to sheep, goat plasma and plasma sediment had larger concentrations of Na and Mg. Sheep serum had a higher Zn content than goat serum, while goat hair had a higher zinc content than sheep hair. Because of the substantial differences between the two species, all of the minerals under investigation are species specific and cannot be generalized from one species to another. The loading and score plot overlap specifically revealed that the highest Cu contents were found in goat blood and serum samples, the highest Cu, Mg, and Zn contents were found in goat blood clot samples, the highest Ca contents were found in sheep plasma and plasma sediment samples, and the highest Ca and Na contents were found in sheep hair samples.

The present results showed different biodistribution in the two species analyzed for all substrates. This different biodistribution in sheep and goats despite being maintained in the same environment may be attributable to the different physiological characteristics and feeding and digestive habits [39]. Sheep are methodical grazers used to eating forage down on the ground. They usually eat short grasses and clover, with good nutritional qualities, and, having their necks naturally curved downward, they mainly ignore trees and shrubs. At the physiological level they also prefer this behavior because they have a distinct groove in the center of the upper lip, called a philtrum [42]. Unlike sheep, goats are browser animals. Goats prefer to eat leaves, twigs and shrubs and often stand up on their hind legs to reach the tops of plants. Goats have been called “intermediate eaters,” which means they have greater dietary flexibility because they graze and graze different species of plants. Their food preferences are broader. Because of their climbing ability, goats can also forage at different spatial levels. Because goats possess a number of physiological adaptations to effectively process a variety of forage qualities. Sheep require vitamins A, D and E. Vitamin A is essential for reproductive activity. Vitamin D is needed to prevent rickets in young animals and osteomalacia in older animals. For example, an excess of copper in the diet of sheep could be toxic, while goats have an important copper requirement [42].

## 5. Conclusions

The two animal species are clearly nutritionally diversified according to species habits. For this reason, the present study aimed to monitor the environmental bioaccumulation of bioactive elements through grazing-fed animals defined as “sentinels”. Considering the close correlation between mineral content in biological sublayers and diet, biomonitoring of blood and keratinized substrates in sheep and goats grazing in the same pasture might be useful to reflexively assess the mineral content of that pasture [43,44]. This is a preliminary study on small ruminants in order to broaden the study of bioaccumulation of bioactive elements in different animal species, explaining the differences in biodistribution between sheep and goats, offering valuable insights for livestock production.

## Figures and Tables

**Table 1:** Analytical parameters for method validation in terms of linearity (R^2^), sensitivity (LOD and LOQ), and accuracy (% of recovery).

Whole Blood Metals Control Level 3					ERM-DB001 (Human Hair)			
Element	R^2^	LOD (mg/Kg)	LOQ (mg/Kg)	Recovery (%)	R^2^	LOD (mg/Kg)	LOQ (mg/Kg)	Recovery (%)
Na	0.9980	1.255	4.142	90.17 ± 1.29	0.9982	1.167	3.851	91.12 ± 1.75
Mg	0.9993	0.051	0.168	96.34 ± 1.51	0.9993	0.045	0.149	96.34 ± 1.41
Ca	0.9989	0.616	2.033	90.56 ± 1.74	0.9988	0.873	2.881	91.57 ± 1.95
Mn	0.9996	0.001	0.003	98.85 ± 0.81	0.9998	0.003	0.010	99.67 ± 0.54
Fe	0.9998	0.010	0.033	98.27 ± 0.66	0.9995	0.018	0.059	98.47 ± 0.82
Cu	0.9985	0.013	0.043	97.68 ± 0.46	0.9985	0.010	0.033	97.25 ± 0.67
Zn	0.9994	0.050	0.165	97.75 ± 0.48	0.9990	0.068	0.224	97.69 ± 0.54

**Table 2:** Mean ± standard deviation SD of comparison between each element in all the analyzed biological substrates of Sheep and Goat (mg/kg of Dry Weight).

Biological Substrate	Ca	Cu	Fe	Mg	Mn	Na	Zn
*Whole Blood*							
Sheep	130.92 ± 10.95 (c) * I **	1.21 ± 0.11 (b)	30.57 ± 4.94 (c) *I*	38.49 ± 4.65 (b)	0.16 ± 0.05 (c) *I*	1794.14 ± 57.06 (c) *I*	19.65 ± 3.41 (c)
Goat	84.26 ± 4.57 (C)	2.29 ± 0.32 (D) *I*	23.78 ± 2.44 (C)	38.76 ± 4.85 (B)	0.11 ± 0.03 (B)	1208.39 ± 55.76 (C)	21.05 ± 3.13 (D)
*Serum*							
Sheep	64.17 ± 5.95 (b) *I*	0.30 ± 0.07 (a)	18.99 ± 2.98 (b) *I*	16.63 ± 3.82 (a)	0.08 ± 0.03 (b) *I*	444.16 ± 20.67 (b) *I*	10.26 ± 3.40 (b) *I*
Goat	42.07 ± 3.49 (B)	0.72 ± 0.10 (B) *I*	15.28 ± 0.94 (B)	15.64 ± 1.69 (A)	0.05 ± 0.02 (A)	419.94 ± 32.67 (B)	8.21 ± 0.43 (B)
*Blood clot*							
Sheep	135.62 ± 11.37 (c) *I*	1.19 ± 0.11 (b)	32.13 ± 4.90 (c) *I*	34.89 ± 5.26 (b)	0.15 ± 0.04 (c)	1790.75 ± 51.82 (c) *I*	17.34 ± 3.70 (c)
Goat	87.78 ± 4.63 (C)	2.35 ± 0.32 (D) *I*	22.67 ± 2.48 (C)	40.54 ± 3.60 (B) *I*	0.12 ± 0.03 (B)	1192.59 ± 48.70 (C)	21.90 ± 2.17 (D) *I*
*Plasma*							
Sheep	74.05 ± 6.16 (b) *I*	0.24 ± 0.07 (a)	15.17 ± 3.12 (b)	16.37 ± 2.96 (a)	0.06 ± 0.02 (a)	530.85 ± 29.97 (b)	13.92 ± 3.67 (bc)
Goat	48.56 ± 4.18 (B)	1.26 ± 0.27 (C) *I*	13.97 ± 3.58 (B)	20.67 ± 3.00 (A) *I*	0.06 ± 0.03 (A)	597.51 ± 17.67 (B) *I*	15.46 ± 2.96 (C)
*Plasma sediment*							
Sheep	77.97 ± 6.30 (b) *I*	0.30 ± 0.07 (a)	18.21 ± 3.39 (b)	19.94 ± 3.37 (a)	0.09 ± 0.03 (b)	541.59 ± 29.81 (b)	17.36 ± 3.43 (c)
Goat	52.47 ± 4.38 (B)	1.45 ± 0.27 (C) *I*	17.08 ± 3.61 (B)	24.44 ± 3.66 (A) *I*	0.11 ± 0.03 (B)	617.70 ± 17.33 (B) *I*	18.25 ± 3.47 (CD)
*Hair*							
Sheep	34.95 ± 7.09 (a) *I*	0.17 ± 0.05 (a)	6.42 ± 0.87 (a)	17.45 ± 5.08 (a)	0.15 ± 0.05 (c)	170.66 ± 13.77(a) *I*	1.85 ± 0.25 (a)
Goat	19.54 ± 4.56 (A)	0.21 ± 0.05 (A) *I*	10.83 ± 1.68 (A) *I*	16.66 ± 3.59 (A)	0.36 ± 0.07 (C) *I*	97.99 ± 4.87 (A)	2.87 ± 0.44 (A) *I*

* Kruskal–Wallis test results for different biological substrates within the same species. Different letters in the same column represent statistically different results (*p* < 0.05). Lowercase letters refer to sheep, whereas maiscules refer to goats. ** Mann–Whitney test results between species for each biological substrate. *I* indicates statistically higher results (*p* < 0.05).

## Data Availability

The raw data supporting the conclusions of this article will be available by the authors on request.
